# Absolute direction in organelle movement

**DOI:** 10.1002/ece3.70092

**Published:** 2024-08-06

**Authors:** Solveig Plomer, Annika Meyer, Philipp Gebhardt, Theresa Ernst, Enrico Schleiff, Gaby Schneider

**Affiliations:** ^1^ Institute of Mathematics Goethe University Frankfurt Frankfurt Germany; ^2^ Faculty of Biological Sciences Goethe University Frankfurt Frankfurt Germany

**Keywords:** absolute angle, biased random walk, correlated random walk, hidden Markov model, movement analysis, turning angle

## Abstract

In movement analysis, correlated random walk (CRW) models often use so‐called turning angles, which are measured relative to the previous movement direction. To segregate between different movement modes, hidden Markov models (HMMs) describe movements as piecewise stationary CRWs in which the distributions of turning angles and step sizes depend on the underlying state. This typically allows for the segregation of movement modes that show different movement speeds. We show that in some cases, it may be interesting to investigate absolute angles, that is, biased random walks (BRWs) instead of turning angles. In particular, while discrimination between states in the turning angle setting can only rely on movement speed, models with absolute angles can be used to discriminate between sections of different movement directions. A preprocessing algorithm is provided that enables the analysis of absolute angles in the existing R package moveHMM. In a data set of movements of cell organelles, models using not the turning angle but the absolute angle could capture interesting additional properties. Goodness‐of‐fit was increased for HMMs with absolute angles, and HMMs with absolute angles tended to choose a higher number of states, suggesting the existence and relevance of prominent directional changes in the present data set. These results suggest that models with absolute angles can provide important information in the analysis of movement patterns if the existence and frequency of directional changes is of biological importance.

## Graphical abstract

In movement analysis, models often use so‐called turning angles, which are measured relative to the previous movement direction. We use hidden Markov models (HMMs) to show that the analysis of absolute angles instead of turning angles can provide important additional information if the existence and frequency of directional changes is of biological relevance. In a data set of movements of cell organelles, goodness‐of‐fit was most often increased for HMMs with absolute angles, and these could therefore be used to investigate important additional properties, such as the switching of movement between periods of different movement directions.

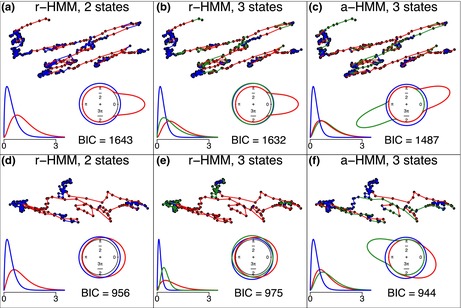

## 1 INTRODUCTION

The description of movement patterns can be important for the understanding of various biological processes. For example, animal movement patterns can describe important functions such as foraging strategies, predation or community interactions (for an overview see, e.g., Bailey et al., 2018; Byrne et al., 2009; Edelhoff et al., 2016; Gurarie et al., 2016). As another example, also the movements of cell organelles can be important indicators of their functional interactions (Block & Jouhet, 2015; Perico & Sparkes, 2018; Shai et al., 2016; Wang et al., 2023).

On the one hand, many animal movement analyses focus on models that aim at partitioning the observed time series into roughly stationary sections (see e.g., Byrne et al., 2009; Edelhoff et al., 2016; Gurarie et al., 2009, 2016; Michelot et al., 2016; Patterson et al., 2017). Theoretical models often use random walks in which the movement direction and step size are drawn from an underlying probability distribution (Bailey, 2019; Bailey et al., 2018; Bartumeus et al., 2008; Codling & Benhamou, 2008; McClintock et al., 2014). Most often used, particularly in animal studies, are so‐called correlated random walks (CRW, Kareiva & Shigesada, 1983; Morales et al., 2004; McClintock et al., 2012). In a CRW, movement is modeled by choosing a random step size and turning angle from a suitable probability distribution in each step. The use of such turning angles indicates that the current movement direction is assumed to depend on the previous movement direction, a behavior which is termed persistence. The resulting models are often useful for the description of animal movements, and CRWs have therefore also been called the “workhorse of modern movement ecology” (Fagan & Calabrese, 2014).

On the other hand, in the context of swimming microorganisms or chemosensitive cells subject to concentration gradients or fluid flows, so‐called biased random walks (BRW), or biased and correlated random walks have been studied, particularly with respect to continuous process limits or the dispersal of populations (Alt, 1980; Codling et al., 2010; Hill & Häder, 1997). In a BRW, an absolute bias in movement direction is introduced, which can be towards a global direction, for example, in the presence of a chemical gradient, or towards a specific point in space, for example, a food source. This bias is assumed to be independent of the previous movement step. Thus, BRWs describe absolute movement direction, or absolute angles, instead of the relative direction, or turning angles. While relative direction in CRWs is measured relative to the previous movement step, absolute direction in BRWs is measured relative to a fixed direction, which can be, for example, the North in animal movement or the x‐axis in arbitrary two‐dimensional space. Here, we measure the absolute angle counterclockwise from the x‐axis.

In the practical analysis of movement patterns that usually deal with animal movements, one therefore typically applies CRWs. One particularly useful method for the segregation of movement patterns into different movement modes is to use a hidden Markov model (HMM) in which the distribution of turning angles and step sizes depends on the hidden state. Such a model has been implemented for straightforward practical application by Michelot et al. (2016) in the moveHMM package in the statistical environment R and generalized to allow for, for example, environmental covariates, attractive forces or more complicated spline models by McClintock and Michelot (2018) in the momentuHMM package.

The main idea in this analysis is to segregate movements into different modes, which usually describe different movement behaviors. For animals, these may include, for example, “flapping, gliding, or soaring in birds; walking, trotting, or galloping in terrestrial quadrupeds” (Conners et al., 2021), activities typically associated with a change in movement speed, whereas changes in movement direction are either considered irrelevant or assumed to occur only rarely during strongly directed movement.

Using HMMs with turning angles, one can efficiently discriminate an observed movement pattern into sections with different movement properties. In particular, such piecewise stationary CRWs with turning angles allow to discriminate between sections of different movement speed. However, sections with similar speed and constant movement direction cannot be discriminated with respect to their absolute movement direction. This is because during strongly directed behavior, turning angles cluster around zero regardless of the absolute movement direction.

Here, we report on a data set of cell organelle movements in which we often observe strong changes in the absolute movement direction which cannot be captured in HMMs that use turning angles. More details on the data set can be found in Plomer, Ernst, Gebhardt, Schleiff, Neininger, and Schneider (2024), where an alternative theoretical model to the random walk is proposed that yields new theoretical insights and an associated method for the detection of change points in direction and speed in movement patterns.

In this data set, it can be of high interest to identify changes in movement direction in addition to changes in speed. This is because, apart from a rather arbitrary force of cytoplasmic streaming (Zawadzki & Fensom, 1986), directed movement can occur along structures of the cytoskeleton (Perico & Sparkes, 2018), for example with the aim of distributing signals or nutrients, potentially in a coordinated manner requiring in part organelle interactions (Block & Jouhet, 2015; Perico & Sparkes, 2018; Shai et al., 2016; Wang et al., 2023). Therefore, a change in direction can represent an important time point for example in the process of nutrient distribution, and the number and size of such changes, potentially even in relation to change points in other organelles, can yield important insights into the mechanisms within the cell.

We therefore compare here the fit of a HMM with turning angles, that is, a classical CRW, with the fit of a HMM with absolute angles in the BRW context. To apply moveHMM with absolute angles instead of turning angles, we propose a simple algorithmic routine. The two HMMs with absolute and turning angles are then applied to tracks from the sample data set, illustrating additional interesting interpretation and increased goodness of fit for the HMM with absolute angles.

## 2 MATERIALS AND METHODS

### 2.1 Data set and dimension reduction

The data set consisted of 41 plastid movement tracks in the root of the plant Arabidopsis thaliana (for details see Plomer, Ernst, Gebhardt, Schleiff, Neininger, & Schneider,  2024). As the data were obtained from microscopy images, all three spatial dimensions can be considered comparable, and their orientation depends on the orientation of the tissue relative to the microscope. However, as reported in Plomer, Ernst, Gebhardt, Schleiff, Neininger, and Schneider (2024), “over 90% of the tracks showed more than 95% of their movement variability in only two dimensions.” We therefore applied principal component analysis to each individual track in order to maximize the movement pattern information captured by the individual components, and considered only the first two dimensions of these movement patterns for the present analysis. The data set of the resulting two dimensional tracks can be found in Dryad (Plomer, Ernst, Gebhardt, Schleiff, & Schneider, 2024).

### 2.2 The HMM with turning angles

**FIGURE 1:**
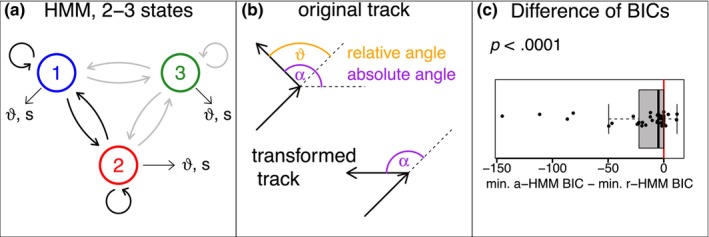
(a) States and transitions of the hidden Markov chain in the HMM with a maximum of three states. If only two states are considered, the blue state usually indicates slow movement and the red state fast movement. For three states, the third state may indicate a third movement speed in the r‐HMM or a‐HMM or a different movement direction in the a‐HMM. (b) Illustration of turning angles ϑ and absolute angles α (upper part) and transformation of the track such that original absolute angles result in turning angles. (c) Differences of BIC values of the chosen a‐HMM and r‐HMM.

Every track was analyzed first with a HMM with turning angles using the moveHMM package (Michelot et al., 2016). That means, movement in the recorded window of length T was assumed to follow a random walk ${\left(X_{t}\right)}_{t=0,\ldots ,T}$. Specifically, the turning angles $\vartheta_{t}$, t=2,…,T (Figure 1b) between successive increments $\left(X_{t}-X_{t-1}\right)$ and $\left(X_{t-1}-X_{t-2}\right)$ were assumed to be independent and von‐Mises distributed, and the step sizes $S_{t}$ at each increment were assumed to be independent and Gamma distributed. The parameters of these distributions may differ between the hidden states in the HMM (Figure 1a).

In order to distinguish this model from the HMM with absolute angles, it will be abbreviated by r‐HMM to illustrate the use of relative, or turning angles. For the r‐HMM, we considered models with one, two, and three hidden states. A maximum of three hidden states was chosen to allow comparison with other studies that aim to distinguish between three main movement modes. For organelle transport, at least two main modes are known as cytoplasmic streaming (Zawadzki & Fensom, 1986) and transport along filaments of the cytoskeleton (Perico & Sparkes, 2018), while the transition between these states or the existence of additional phases remains unclear. We therefore chose a maximum of three states to prevent overinterpretation of the behavior during the relatively short organelle tracks.

### 2.3 The HMM with absolute angles

We then analyzed all tracks with a modified HMM in which the absolute angle, $\alpha_{t}$, of a track increment relative to the *x*‐axis was considered instead of the turning angle $\vartheta_{t}$ (Figure 1b). Note that this analysis is mathematically different from the one with turning angles because with absolute angles, all angles are measured with respect to the same reference, while in the r‐HMM, every angle is measured with respect to the previous increment, whose direction varies with time. Technically, the corresponding BRW $\left(X_{t}\right)$ can be described by an arbitrary starting point and increments $\left(X_{t}-X_{t-1}\right)=\left(S_{t}\cos\left(\alpha_{t}\right),S_{t}\sin\left(\alpha_{t}\right)\right)$, where the absolute angles $\alpha_{t}$ measured relative to an absolute direction, that is, the first principal component, were assumed to be independent and von‐Mises distributed and $S_{t}$ independent and Gamma distributed. The parameters of the distributions may differ between states. This model will be abbreviated a‐HMM. Again, we applied a‐HMMs with one, two, and three states. Biologically, the third state in the a‐HMM allows for, either, a third state with medium speed or a discrimination of a second main movement direction.

In order to perform this analysis, we transformed the original track such that the absolute angles of the original track were represented as turning angles in the transformed track (see Figure 1b). The R‐code for this transformation, which does not require additional R‐packages, is given in Code S1. Note that this transformation does not alter the information given in the original track. Instead, it represents a simple preprocessing step that enables plugging in the transformed track directly into the existing routine. The analysis can in principle also be performed with the moveHMM routines if one artificially replaces the automatically derived turning angles by absolute angles that one derives separately. The respective code can also be found in Code S1.

### 2.4 Parameter estimation and choice of starting parameters

In order to maximize the probability of finding the global maximum of the likelihood function, we used a combination of different starting parameters for the HMM fit. As starting values for the HMMs with one state we used all combinations of the location measure $\mu \in \left\{0,\pi \right\}$ and concentration measure $\kappa \in \left\{1,5\right\}$ for the von‐Mises distribution combined with all combinations of the expectation and standard deviation $\mu_{\Gamma }\in \left\{0.1,0.4\right\}$ and $\sigma_{\Gamma }\in \left\{0.1,5\right\}$, respectively, for the Gamma distribution. In the HMMs with two states, we used all combinations of starting values $\mu_{1}\in \left\{0,\pi /8\right\}$, $\kappa_{1}\in \left\{1,2\right\}$, $\mu_{2}\in \left\{0,\pi /8\right\}$, $\kappa_{2}\in \left\{1,2\right\}$ for the von‐Mises distributions of states 1 and 2, respectively, and starting values $\mu_{\Gamma }^{\left(1\right)}\in \left\{0.1,0.4\right\}$, $\sigma_{\Gamma }^{\left(1\right)}\in \left\{0.1,0.5\right\}$, $\mu_{\Gamma }^{\left(2\right)}\in \left\{0.1,0.8\right\}$, $\sigma_{\Gamma }^{\left(2\right)}\in \left\{0.1,0.5\right\}$ for the expectation and standard deviation of the Gamma distribution of states 1 and 2, respectively. In the HMMs with three states, we used all combinations of starting values $\mu_{1}=0$, $\kappa_{1}=1$, $\mu_{2}\in \left\{0,\pi /2\right\}$, $\kappa_{2}\in \left\{1,2\right\}$, $\mu_{3}\in \left\{0,\pi /2\right\}$, $\kappa_{3}\in \left\{1,2\right\}$ for the von‐Mises distributions of states 1−3, respectively, and starting values $\mu_{\Gamma }^{\left(1\right)}\in \left\{0.1\right\}$, $\sigma_{\Gamma }^{\left(1\right)}=0.1$, $\mu_{\Gamma }^{\left(2\right)}\in \left\{0.1,0.8\right\}$, $\sigma_{\Gamma }^{\left(2\right)}\in \left\{0.1,0.5\right\}$, $\mu_{\Gamma }^{\left(3\right)}\in \left\{0.1,0.4\right\}$, $\sigma_{\Gamma }^{\left(3\right)}\in \left\{0.1,0.5\right\}$ for the expectation and standard deviation of the Gamma distribution of states 1–3, respectively. For each number of states, we separately chose the resulting HMM with the maximal likelihood, where we ignored fits resulting in warning messages.

### 2.5 Choosing the number of hidden states

In order to choose between the resulting HMM fits with one, two, and three states, we first discarded from the analysis such fits that yielded degenerated states which were only observed for a single time instant. The remaining models were then compared using the BIC. The BIC was chosen in order to prevent overestimation of the number of states. In a small simulation with 10 r‐HMMs for one, two, and three states, respectively, and fitted r‐HMMs with 1–4 states, we compared the AICs and BICs derived for the different numbers of states, investigating the criteria AIC or BIC for the choice of the number of states. In these simulations, the AIC most often overestimated the number of states, while the BIC was optimal for the correct number of states (Table 1).

**TABLE 1:** True (rows) and estimated (columns) numbers of states in simulated r‐HMMs using AIC or BIC criteria in a sample simulation with 10 tracks for each true number of states.

True/estimated	AIC				BIC			
	1	2	3	4	1	2	3	4
1	0	1	9	0	10	0	0	0
2	0	0	9	1	0	10	0	0
3	0	0	1	9	0	0	10	0

## 3 RESULTS

### 3.1 Model choice

For most of the tracks, the chosen number of states was comparable for the r‐HMM and the a‐HMM: of 41 tracks, 12 were modeled with one state, 14 tracks with two states and three tracks with three states in both models.

For only 12 tracks, the chosen number of states differed between the models. In all of these cases, the r‐HMM chose fewer states than the a‐HMM: seven tracks showed three states in the a‐HMM and two states in the r‐HMM, one track showed three states in the a‐HMM and one state in the r‐HMM, and four tracks showed two states in the a‐HMM and one state in the r‐HMM.

Interestingly, although the chosen number of states was usually comparable for the a‐HMM and the r‐HMM, the respective best BIC was smaller for the a‐HMM in 35 out of 41 tracks (Figure 1c, *p* < .0001, Wilcoxon test), suggesting a closer description of the present data set with the a‐HMM with absolute angles. Note that highly similar results were obtained when using a wrapped Cauchy distribution instead of a von‐Mises distribution in the HMM fitting for both absolute and relative angles (data not shown).

### 3.2 Absolute angles complement interpretation of models with turning angles

A more detailed look at the fitted a‐HMMs and r‐HMMs for two tracks (Figure 1a–c for track 1 and d–f for track 2) reveals interesting additional aspects with respect to the interpretation of the fitted hidden states. First, we note that models with two states are usually similar for the a‐HMM and the r‐HMM, discriminating in the present data set between fast and slow movement (red and blue states, respectively, Figure 1a,d, results for a‐HMM not shown). Second, if r‐HMMs with three states were chosen, the r‐HMM usually discriminated between three different speeds of movement. To illustrate this, panels b and e indicate differences in the distributions of s, while the distributions of ϑ are only either approximately uniform or concentrated around zero for fast directed movement (red state in Figure 1b). In contrast, the a‐HMM with three states, which shows a smaller BIC here, does prefer to divide the state with fast directed movement into two states with the same fast movement but opposite movement directions (Figure 1c,f).

**FIGURE 2:**
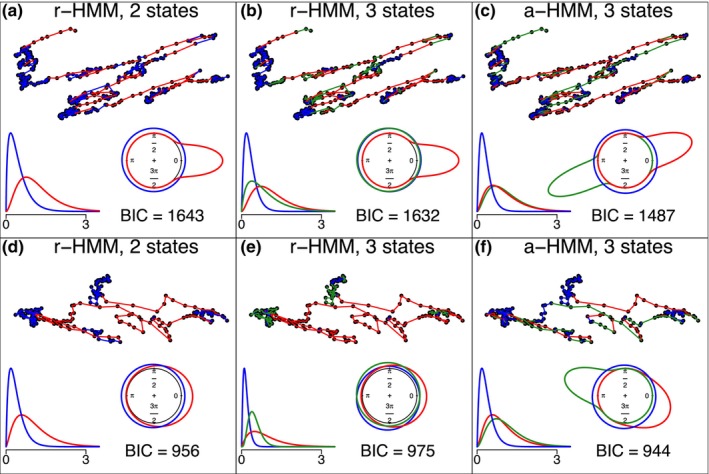
(a)–(c) Analysis of track 1, with r‐HMM with two states (a), three states (b) and a‐HMM with three states (c). (d)–(f) Analysis of track 2, with r‐HMM with two states (d), three states (e) and a‐HMM with three states (f). (a)–(f): States are color coded. The Viterbi path of the track in the corresponding model is shown in the upper part. Lower part indicates the fitted von‐Mises and Gamma distributions of angles and step sizes, respectively.

**FIGURE 3:**
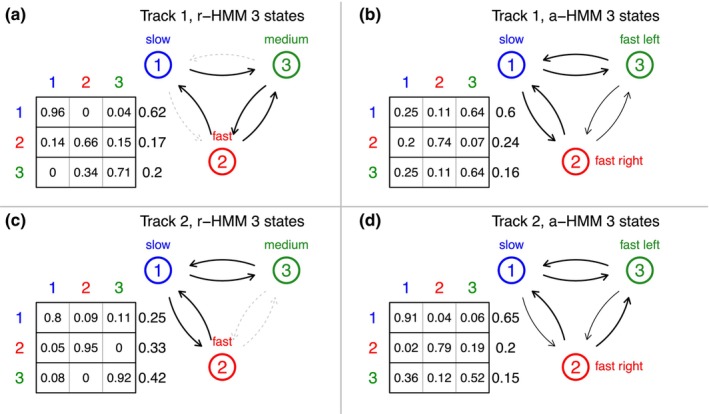
Transition probabilities for the r‐HMM and a‐HMM with three states for the tracks from Figure 1 indicated by arrows of different widths. The column to the right of each table indicates the fraction of time spent in the respective state. If no transition was observed between two states, the corresponding arrow is dashed.

Also, the transition probabilities of individual tracks may provide interesting information on the potential function of the respective organelle. Figure 3 indicates the estimated transition probabilities of the tracks shown in Figure 2 for the r‐HMM (left) and the a‐HMM (right) for three states. For convenience, the states are abbreviated here as “slow,” “fast,” and “medium” in the r‐HMM and “slow,” “fast left,” and “fast right” in the a‐HMM. These names are, however, only meant relative to the respective track and cannot be considered absolutely comparable across tracks, especially because different tracks may correspond to different functions such as transport or interaction.

For the r‐HMM, we observe interesting differences between the tracks: In track 1 (Figure 3a), no transitions are observed between the slow state and the fast state, indicating that acceleration occurs slowly. In addition, neither are transitions observed between the state of medium speed and the slow state, indicating that deceleration occurs abruptly in this organelle. In track 2 (Figure 3c), all transitions between the slow state and the other states are observed, but here, no transitions are observed between the fast state and the state of medium speed in either direction. This might indicate potentially different functions for the two states with different speeds in this track.

For the a‐HMM, patterns naturally differ from the r‐HMM due to different state interpretations, but they are similar for the two organelles (Figure 3b,d). In particular, all possible transitions between the states are observed, which means that transitions between the two fast states with opposite directions are also observed. Thus, the organelles can show rapid full turns in the direction at high speed, although these transitions expectedly tend to have smaller probability. The mean number of time steps spent in each state before leaving the respective state is shown in Table 2.

**TABLE 2:** Mean number of time steps spent in each state before leaving the respective state as shown in Figure 3.

	r‐HMM			a‐HMM		
	1	2	3	1	2	3
Track 1	21.7	2.9	3.4	6.8	3.8	2.7
Track 2	4.9	11.2	18.2	10	4.8	2.1

Thus, for the present data set, both, r‐HMM and a‐HMM can yield interesting insights into the movement patterns. While the r‐HMM focuses on changes in speed, the a‐HMM offers the additional property to distinguish between sections of different directions during periods of highly directed movement. The BIC values, which typically found the correct number of states in our simulations, suggested an advantage of the a‐HMM. This indicates that changes in the absolute movement direction seem to be a prominent feature in the present data set. This is also supported by the observation that the r‐HMM has a tendency to choose fewer states than the a‐HMM does. It indicates that it seems to be preferable to add an additional state in the a‐HMM, which is able to represent a different absolute movement direction, rather than adding an additional state in the r‐HMM, which would typically represent an additional state with a different movement speed.

Note that due to the preprocessing of the data by principal component analysis, a higher degree of movement within the direction of the *x*‐axis, which corresponds to the first principal component, can be expected. This is why we have chosen the *x*‐axis here as the absolute reference direction. This direction can, however, be chosen arbitrarily. When the method is applied to animal movements for example, the angle from the north may be another standard reference. Also, one needs to keep in mind that results may depend on the distributional assumptions used in the analysis (Bailey & Codling, 2021; Choules & Petrovskii, 2017). However, in the present context, the use of the von‐Mises and the wrapped Cauchy distribution yielded highly comparable results, and we believe that the exact choice of the distributional assumption may be less important if the overall shape of the distribution remains similar and is thus capable of capturing the observed phenomena.

## 4 DISCUSSION

In this contribution, we aimed to show that for movement analysis in certain data sets such as the one presented here, it can be advantageous to incorporate models with absolute movement direction, or BRWs, instead of or in addition to the widely used CRWs. While the classical animal movement analysis focuses on the segregation of sections with different movement speeds and therefore uses turning angles, we propose to extend this approach to the analysis of absolute angles. We have presented a simple preprocessing algorithm in R that transforms the original track into a track in which original absolute angles are represented as relative angles, allowing direct application of the moveHMM package to compare r‐HMMs with turning angles to a‐HMMs with absolute angles.

The analysis of our data set of cell organelle movements suggests that the proposed a‐HMM model with absolute angles can provide a closer representation of some data sets than the r‐HMM with turning angles. Specifically, in the present data set, adding a state in the a‐HMM, which corresponds to an additional movement direction, seems to be advantageous over adding a state in the r‐HMM, which typically represents a state with an additional movement speed. Biologically, changes in the movement direction of cell organelles can for example indicate that the organelle is switching its attachment between different filament structures, and the frequency and potential coordination of directional changes can therefore be important indicators of functional mechanisms and their variability across organelles as well as potential interactions in signaling pathways and nutrient distribution within the cell.

Note in this context that only little is known about the movement type and function of cell organelles within the cell. It is therefore highly interesting to capture the prominent features of the movement as such. The prominent changes in movement direction seem to be inherent properties of the tracks and not directly imposed by the environmental structure. Although the intracellular space is crowded by the abundance of multiple organelles and cytoskeletal structures, the available movement space for one organelle is typically much larger within the cell also because of the dynamic of the cellular interior. During the whole recording, the organelles, which have a diameter of about 2–5 micrometers, move for a maximal distance of up to about 10–20 micrometers within a cell of size about 100×30×30 micrometers, showing a high number of directional changes during this process. As the organelles move between the cell border and the inner vacuole, which takes a considerable part of the cell volume, a certain reduction to a two‐dimensional movement pattern may be plausible as observed in the present data set. However, the frequency of observed directional as well as speed changes is much higher than would be expected simply from limitations in the cellular environment.

Thus, approaches with absolute angles can be advantageous in such cases in which directional changes during highly directed behavior are frequent and biologically interesting. If the explicit direction, or the change in direction, or the frequency of directional changes does not seem relevant, for example when aiming to differentiate between movement modes that are associated with different movement speeds, models with absolute angles may not provide considerable advantages. For example, when animals are moving between different foraging locations, the specific movement direction may not necessarily be relevant. In other cases, one may not observe directional changes at all or different patterns such as circular‐like movement. In such cases, the widely used CRWs or also biased and correlated random walks are plausible and useful candidate models, and the additional incorporation of environmental covariates or attractive forces as proposed by McClintock and Michelot (2018) can provide important biological insights.

One drawback in the analysis based on HMMs is the necessity to pre‐specify the number of states. This requires precise biological pre‐knowledge as well as the existence of the pre‐assumed states in each observed finite movement pattern. While a comparison of HMMs with different numbers of states as performed here may be one way to deal with this issue, one could also apply change point analysis to investigate changes in the movement direction and speed. In the present application context, Plomer, Ernst, Gebhardt, Schleiff, Neininger, and Schneider (2024) have developed methods for bivariate change detection both in the context of the BRW and of a newly proposed movement model in which a track sticks even more closely to a straight line than a BRW. As another possibility for future research, one might also consider regularized estimation techniques, which can be applied to trajectories, that is, movements (Steyer et al., 2023; Stöcker et al., 2023) and have been used successfully for the detection of an unknown number of change points (Otto & Steinert, 2023).

In summary, we believe that it can be of high interest to complement existing approaches in applied movement analysis typically focusing on turning angles and CRWs by models with absolute angles, or BRWs. This can be of particular relevance in the analysis of movement patterns when the existence and frequency of directional changes can yield interesting biological observations. The application as presented here is straightforward and can provide interesting additional insights into the dynamics of biological movement patterns.

## AUTHOR CONTRIBUTIONS

**Solveig Plomer:** Data curation (equal); formal analysis (supporting); supervision (supporting); writing – original draft (equal). **Annika Meyer:** Data curation (equal); formal analysis (lead); investigation (equal); visualization (equal). **Philipp Gebhardt:** Data curation (equal); investigation (equal); writing – review and editing (supporting). **Theresa Ernst:** Conceptualization (supporting); data curation (equal); investigation (supporting); writing – review and editing (supporting). **Enrico Schleiff:** Funding acquisition (lead); supervision (supporting); writing – review and editing (supporting). **Gaby Schneider:** Conceptualization (lead); formal analysis (equal); funding acquisition (lead); project administration (lead); supervision (lead); visualization (equal); writing – original draft (lead); writing – review and editing (lead).

## CONFLICT OF INTEREST STATEMENT

The authors declare that they have no conflict of interest.

## Supporting information


Code S1.

## Data Availability

The data set of 41 tracks projected onto the first two principal components is provided in Dryad (Plomer, Ernst, Gebhardt, Schleiff, & Schneider, 2024). The URL is https://doi.org/10.5061/dryad.5hqbzkhfd. Tracks numbered 1 and 2 in the manuscript are the tracks with IDs 102 and 520, respectively. The analysis code uses the publicly available moveHMM package in the statistical software environment R extended with the algorithmic transformations as described in the manuscript.
